# Optophysiological Characterisation of Inner Retina Responses with High-Resolution Optical Coherence Tomography

**DOI:** 10.1038/s41598-018-19975-x

**Published:** 2018-01-29

**Authors:** Irina Erchova, Alexandre R. Tumlinson, James Fergusson, Nick White, Wolfgang Drexler, Frank Sengpiel, James E. Morgan

**Affiliations:** 1 School of Optometry and Visual Sciences, Cardiff University, Cardiff, United Kingdom; 2 School of Biosciences, Cardiff University, Cardiff, United Kingdom; 3 Ophthalmology, University Hospital of Wales, Cardiff, United Kingdom; 4 Center for Medical Physics & Biom Eng, Medical University Vienna, Vienna, Austria; 5 Zeiss Meditec, Dublin, California USA

## Abstract

Low coherence laser interferometry has revolutionised quantitative biomedical imaging of optically transparent structures at cellular resolutions. We report the first optical recording of neuronal excitation at cellular resolution in the inner retina by quantifying optically recorded stimulus-evoked responses from the retinal ganglion cell layer and comparing them with an electrophysiological standard. We imaged anaesthetised paralysed tree shrews, gated image acquisition, and used numerical filters to eliminate noise arising from retinal movements during respiratory and cardiac cycles. We observed increases in contrast variability in the retinal ganglion cell layer and nerve fibre layer with flash stimuli and gratings. Regions of interest were subdivided into three-dimensional patches (up to 5–15 μm in diameter) based on response similarity. We hypothesise that these patches correspond to individual cells, or segments of blood vessels within the inner retina. We observed a close correlation between the patch optical responses and mean electrical activity of the visual neurons in afferent pathway. While our data suggest that optical imaging of retinal activity is possible with high resolution OCT, the technical challenges are not trivial.

## Introduction

Optical Coherence Tomography (OCT) derives high resolution spatially distinct images from the interferometric analysis of tissues probed with low coherence laser light, similar to ultrasound^1,2^. It is widely used for clinical diagnostic imaging of eye diseases^3,4^ and other structures of biomedical interest such as skin and colon where changes in tissue thickness can be quantified at near cellular resolution^5,6^. The contrast sensitivity of OCTs using broad spectral light sources is close to that required to detect neuronal light scattering, birefringence, and structural changes associated with action potentials as reported in pioneering work by Cohen^7–11^. However, Cohen’s work was based on tissue analysed *in vitro* and relied on signals from large individual neurons. The ability to register a reliable optical responses, *in vivo*, from retinal neurons within a tissue volume remains a matter of considerable debate.

The use of OCT to detect optical changes associated with activity has been termed “optophysiology”^12^. *In vivo* light-evoked responses have been detected with OCT in cat visual cortex^13,14^ that were comparable in amplitude to brain intrinsic signal imaging^15^. In the retina, transient optophysiological responses have been detected *in vitro* in the outer retina (photoreceptors) in response to light in the frog^16^ and rabbit^12^. Comparable *in vivo* responses have been reported in rat^17^, and human^18^. More recently Hillmann *et al*.^19^, using a different OCT technique, evaluated *in vivo* phase data from a parallelized and computationally aberration-corrected OCT and obtained spatially and temporally resolved changes in the optical path length of the photoreceptor outer segment in the human eye.

These studies report optical changes at the organ level or, in the context of retinal imaging, within a given retinal layer. Isolating these responses at the cellular level outside of the photoreceptor layer has proved elusive. Mihashi *et al*.^20^ reported reflectance changes in the cat retina following electrical stimulation of the chiasm that were completely suppressed by tetrodotoxin, suggesting a contribution of the inner retinal layers to the intrinsic signals. In *ex vivo* frog’s retina Li *et al*.^21^ reported wide-spread, small, and inconsistent optical transients in the inner plexiform and retinal ganglion cell (RGC) layer. In macaque retina *in vivo*, Suzuki *et al*.^22^ reported a similar widespread pattern of activation in inner retina and attributed it entirely to blood flow. Moayed *et al*.^23^ successfully detected fast transient light-evoked signals from the chicken retina (*in vivo*), which were strongly linked to membrane potential changes. Most recently, Morimoto *et al*.^24^ suggested that the inner retinal responses obtained in *ex vivo* cat retinas were specific to spatial frequencies of presented gratings, and reflected neural activity rather than blood oxygenation. The neuronal component increased with increasing wavelength of the light source, peaking at the lowest spatial frequency (0.05 cycles/degree) and attenuated at higher spatial frequencies, consistent with known RGC response properties. However, the time course of optophysiological response differed from known electrophysiology, suggesting the confounding effects of retinal metabolic activity on the OCT signal.

Collectively, these studies make a strong case for the use of OCT in the optical detection of neuronal activity. Since the axial resolution of broad spectrum OCT approximates cellular dimension, we reasoned that the derivation of optophysiological signals at this scale would overcome the confounding effects of different response polarities within a tissue layer.

To test this, we undertook high-resolution OCT analyses in tree shrews (Tupaia belangeri), an excellent non-primate model of the human retina. We eliminated the confounding effects of eye movements using whole animal paralysis under deep anaesthesia. The OCT was a custom built high resolution device with long wavelength 1040 nm light source (invisible to humans and most of animals) to maximise the neuronal component of the OCT signal (Supplemental materials, Figures S1, S2) and avoid possible visual stimulation with OCT measurement light. We used advanced cluster analysis techniques to isolate the *in vivo* optophysiological responses from neuronal groups within the retinal ganglion cell layer.

## Results

**Figure 1:**
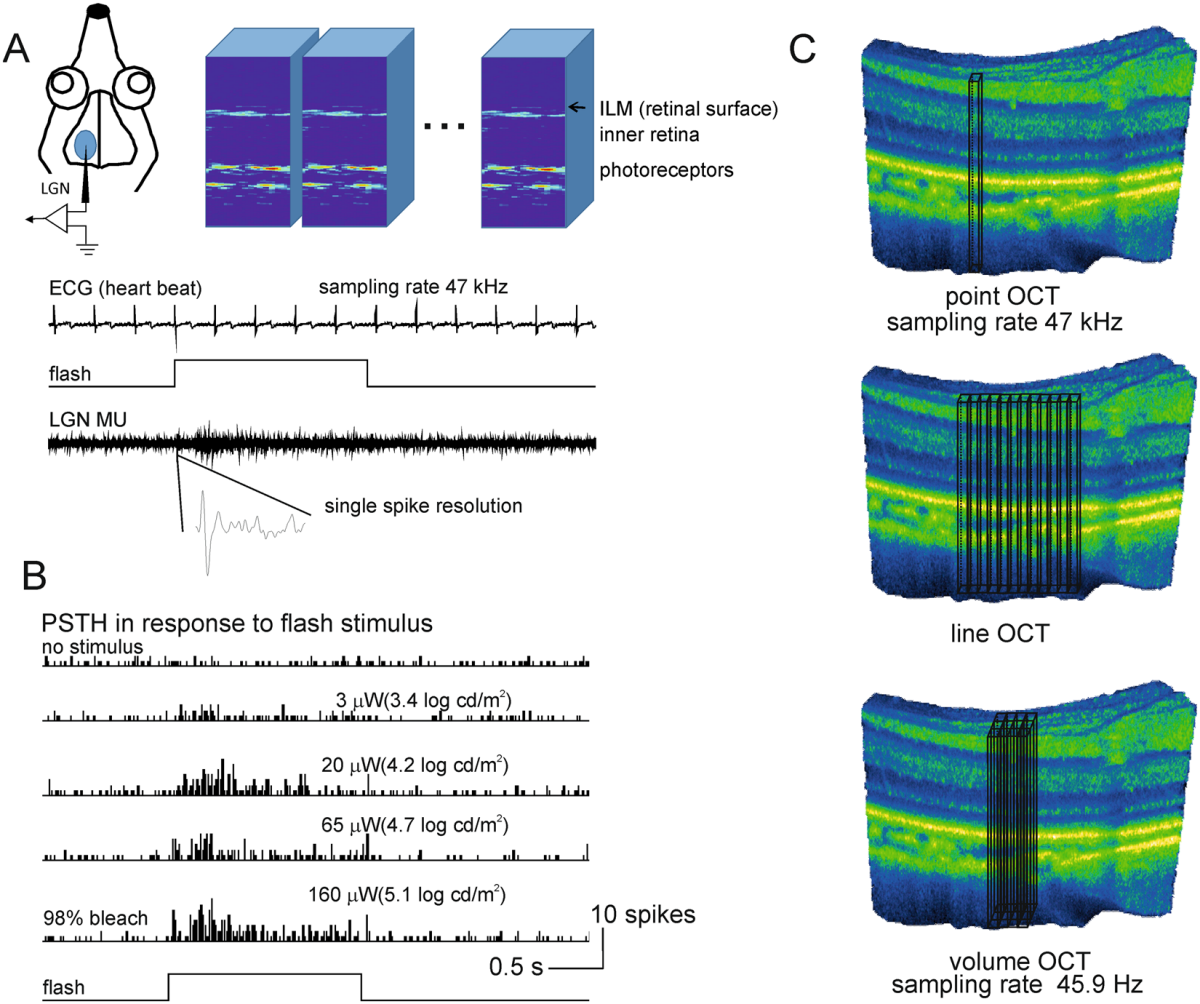
Experimental Design. Tree shrews were anaesthetised; the retina was exposed to light stimuli and retinal movements minimised (pupils were dilated; eye muscles were paralyzed, and the animal artificially ventilated). (**A**) A single extracellular electrode was inserted into the left lateral geniculate nucleus (LGN) to monitor activity of axonal projections of RGCs from the contralateral (right) eye in response to light stimuli (LED flashes or high contrast moving gratings of various spatial frequencies). The aggregate receptive field of LGN multiunit activity (MUA) was found using audio monitor, movable computer screen, and OCT attached stimulating LED light, focused at the same point on the retina as the OCT measurement beam (to allow selective stimulation of the imaged retinal area). A small area of retina within the receptive field of LGN MUA (usually in the middle retina 32 by 32 pixels, approximately 25 by 25 μm^2^, see Fig. S2) was then imaged repeatedly for 5 s with 47 kHz for a single A-line of OCT. The ECG (heart beat), stimulus, and LGN MUA were recorded simultaneously with the same sampling frequency. ILM is the inner limiting membrane.(**B**) LGN MUA in response to flashes of four different levels of brightness (2 s stimulus) ranging from threshold for RGC activation to 98% bleach. Post-time-stimulus histograms (PSTH) with 20 ms bin accumulated over three trials show reliable responses correlated with flash intensity. (**C**) Schematic showing the three different types of OCT scans we performed. Point scans were carried out at a maximum sampling frequency of 47 kHz. For line scans sampling rate was reduced proportionally to the length of line, for volume scans 32 × 32 pixels sampling rate reduced to 45.9 Hz.

We obtained functional retinal OCT signals from 8 adult tree shrews. We undertook three OCT scan protocols across the depth of the retina: point scans at 47 kHz (sampling rate is limited by the speed of camera), line scans at various line lengths (sampling rate is additionally limited by the length of line), and volume scans of 32 × 32 pixels at 45.9 Hz (sampling rate is additionally limited by the area of scan) (Fig. 1). For point scans the retina was imaged repeatedly for 5 s at 47 kHz (the maximum allowed by our system) for a single OCT A-scan. At the same time, afferent multi-unit activity (MUA) in the lateral geniculate nucleus (LGN) was recorded at 47 kHz, a sampling rate sufficient for single-spike resolution.

We used the inner limiting membrane (ILM) retinal surface, and the bright bands in the outer retina, to align images across trials. The greatest challenge with respect to alignment was residual movement of the retina due to cardiovascular response. While the imaged retinal region of interest was very consistent between trials (see supplemental videos), the micro movements due to blood circulation made point or line imaging of the same area across trials virtually impossible. However, with small volume imaging we were able to compensate micro movements by image registration and re-alignment during image processing. In the following we therefore focus on our volume imaging data. The use of volume data required a reduction in the sampling rate by a factor of 1000 to 45.9 Hz, thereby reducing the temporal resolution of the optical signal from the equivalent of single spikes to a mean firing rate.

**Figure 2:**
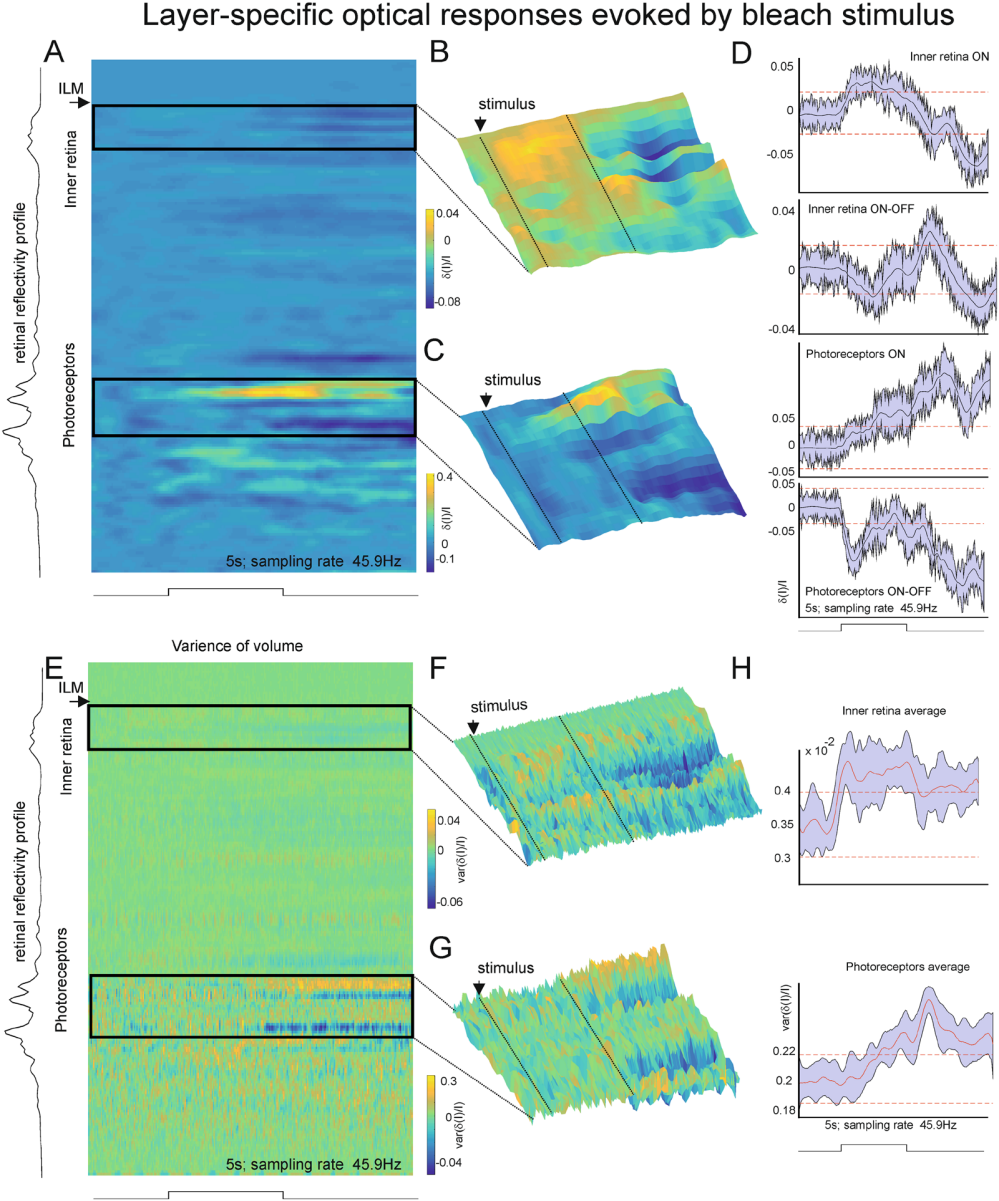
Retinal responses to bright flash across layers. (**A**) Normalised mean of evoked response averaged across one recording area and several trials of the bleach flash stimulus (the time required to recovery of photoreceptors is calculated in Methods section). The Y-axis represents retinal layers from the surface (ILM, inner limiting membrane, top) to deep retina, X axis represents time of recording. Each point in time represents the averaged activity of one spatial volume over 3 trials separated by 60 minutes. Both inner retina and photoreceptor layers show stimulus-locked pattern of activation magnified in **B** and **C** (regions boxed in **A**). **A** and **B** share the same colour scale. (**B**) Magnified surface plot of stimulus locked response of inner retina. The ON and OFF stimulus-locked responses are clearly visible. (**C**) Surface plot of photoreceptor’ responses. The average responses of this area to 3 presentation of the stimulus are shown in (**D**) Each trace represents average activity of retinal area in a narrow layer band. Two upper plots show examples of ON and ON-OFF responses in inner retina and two lower plots show examples of ON and OFF photoreceptor’ responses. The traces are presented with max error (max observed deviation of signal from the mean for a given time point). The red dashed lines represent the significance levels of 3 standard deviation from the trials recorded without a stimulus presentation. (**E**) Normalised variance of evoked response originating from one recording area and accumulated over several trials of the bleach flash stimulus (similar to **A**). **F** and **G** represent re-scaled surface plots of data in **E** (**E** and **G** share the same colour scale). The examples show stimulus-locked change in response variability in inner retina and in photoreceptor layers. (**H**) The stimulus-locked accumulated variance averaged across all recorded areas (10) in several animals (6). The running error represent max observed deviation of signal from the mean in a given time point. The red dashed lines represent the significance threshold of 3 standard deviation from the trials recorded without stimulus presentation as before. Though this noise measure represents a combination of biological noise (due to variability in cell responses) and instrumental noise that cannot be removed, there is a noticeable difference in the stimulus locked responses of inner retina and photoreceptor’ layers.

Typical retinal responses to a 98% bleach flash stimulus are shown in Fig. 2A–D (calculated across 3 trials in the same area). The time between stimuli presentations was up to 50 minutes to allow re-adaptation (see methods). The background OCT signal was measured in the dark over 5 s (the same length as an individual visual stimulus) and several repetitions were averaged prior to the beginning of visual stimulation. We used these measurements to calculate a background optical signal (no stimulation) and a threshold of 3 standard deviations for response detection. The inner retinal responses were generally much smaller compared to those from the photoreceptor layer and showed considerable regional variability. Figure 2B demonstrates ON and ON-OFF stimulus related activity in the retinal ganglion cell layer (quantified in Fig. 2D). The photoreceptor response was similar to that reported by others (Fig. 2C,D).

We applied the same visual stimulation and OCT measurement protocol to 10 different areas in 6 tree shrews. The combination of area responses required image processing to overcome the variation in tissue layer thickness (due to inter-subject variability and differences in location relative to the ONH (see Supplementary Figure 2). Analysis of the mean variance of the responses in inner retina and in photoreceptor layer (Fig. 2H), demonstrated stimulus induced differences. In the inner retina, the variance increased rapidly after stimulus onset and decreased partially after stimulus offset. By contrast, in the photoreceptor layers the variance was minimal at stimulus onset and increased gradually with the duration of the stimulus. Clear signals were seen (both in the mean response and the variance of response) from the photoreceptor outer and inner segments and, to a lesser extent, the retinal ganglion cell layer. All subsequent analyses focused on responses from the RGC layer.

**Figure 3:**
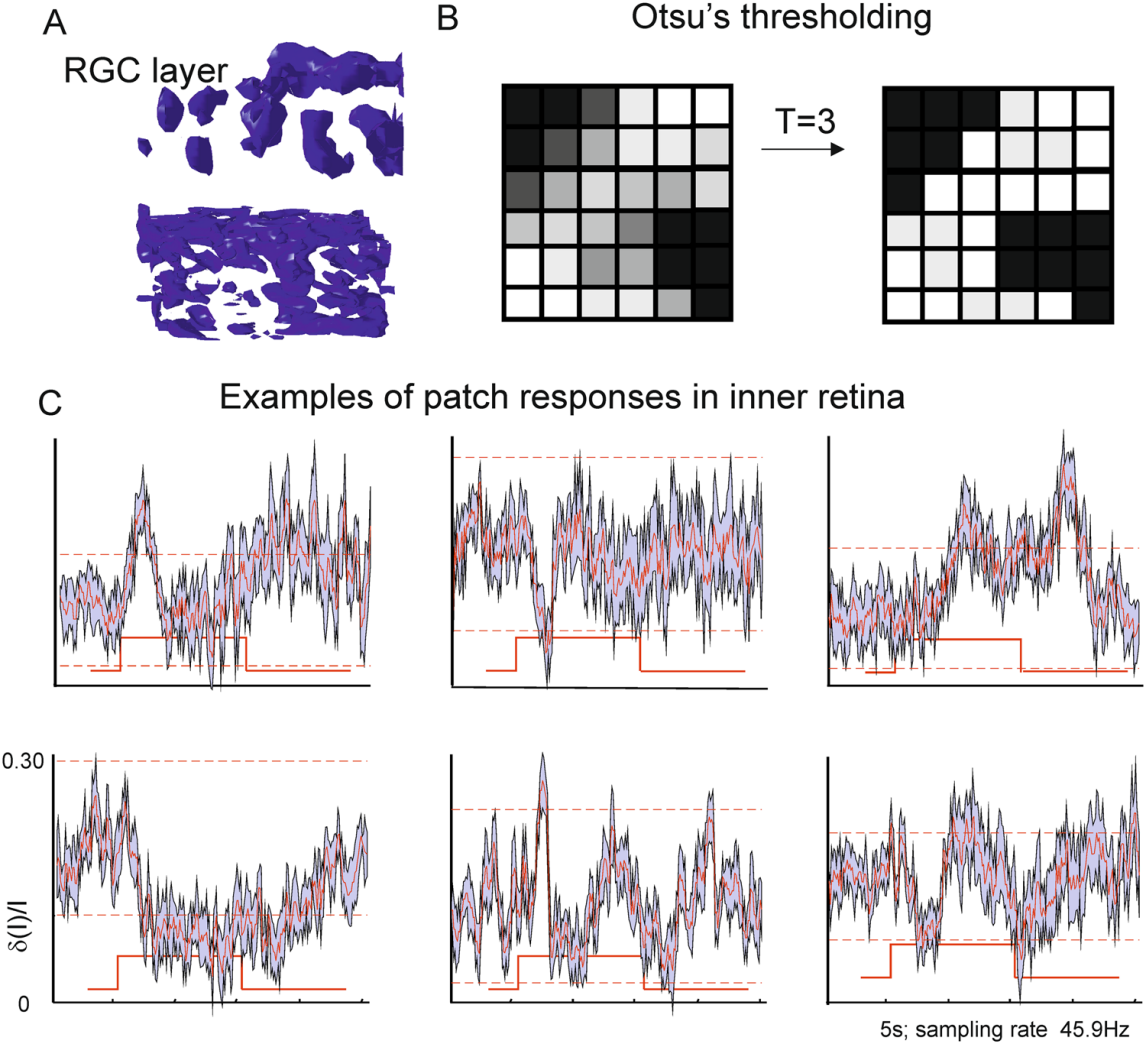
Patch segmentation. (**A**) Equal surface image of the RGC layer (top) and one of the bright regions in the outer retina (bottom). The RGC layer shows clear separation of the signal into individual patches. (**B**) Otsu’s^43^ thresholding (parameter T = 3) applied to separate individual patches. We were able to separate 78 patches in 10 volumes from 6 tree shrews. (**C**) Examples of individual patch responses. All traces represented as an average across all trials and all pixels within a given patch (60–120 traces depending on the patch). The response is given with max running error (max observed deviation of signal from the mean in a given time point); the threshold level of 3 standard deviations from the background is shown as red dashed line.

In order to determine whether the responses were clustered at spatial scales that would be expected for responses originating from individual RGCs, we subdivided the regions of interest in the inner retina into “patches” based on the intrinsic similarity of pixel responses to the visual stimuli (see Methods, Fig. 3). To distinguish “cluster” signals from “speckle” - a noise pattern created by tissue micro-movements, the multiplicative speckle noise was filtered out during image processing. Since our measurements were performed on a spatial scale close to the optical resolution of the system where influence of OCT speckle could be rather complex (having temporal dynamics similar to visually evoked responses), we compared the distribution of our “functional” patches with the distribution of “speckle” patches from the raw images (Fig. S4). All “functional” patches that had one dimension smaller than 4 pixels were discarded, as they could (potentially) reflect complex “speckle” noise. This restriction prevented us from analysing dynamics in the nerve fibre layer (containing axons of RGC cells) because the thickness of axons is below the optical resolution of the system and signal from this layer could be confounded by speckle noise.

We analysed 78 patches from 10 areas. Only 42 individual patches showed stimulus-evoked responses 3 standard deviations above baseline and were included in further patch analyses. Several types of stimulus locked responses were detected. We subdivided them in ON only (34 patches), OFF only (5 patches), and ON and OFF responses (3 patches). The polarity of the responses, delays and time courses were specific to each activated region. The typical size of a patch was 5–15 pixels in diameter (10 μm^3^) corresponding to dimensions of individual RGCs and minor blood vessels within the inner retina.

For the brightest flash (5.1 log cd/m^2^, 98% bleach of cone receptors, based on human opsin dynamics), the average change in contrast was 31 ± 4% for the ON response and 23 ± 6% for the OFF response, with individual values ranging from 2.2% to 62% (see Fig. 3C for examples). Quantitative analysis and comparison with simultaneously obtained electrophysiological recordings indicated three distinct response kinetics.

**Figure 4:**
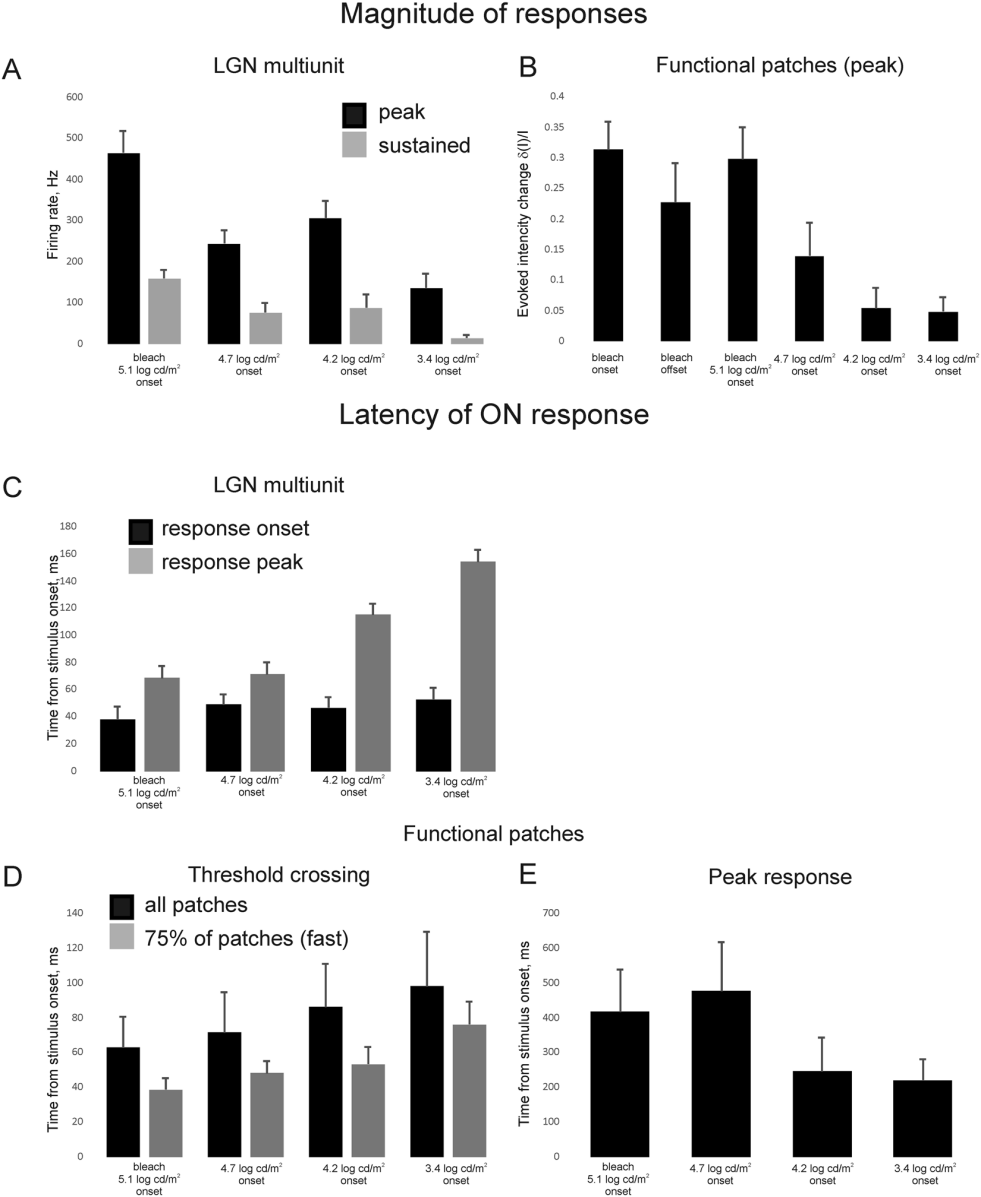
Summary of the patch analyses. (**A**) Average magnitude of the response of LGN multiunit (both for initial peak response and delayed sustained response). (**B**) Average peak response of functional patches. On average brighter flashes evoked larger responses. Most of the patches responded to ON phase of the flash. The OFF responses were present only in less than 15% of patches and were on average smaller. Detailed analysis was therefore restricted to the ON responses. (**C**) Average latency of LGN multiunit ON response (both threshold crossing and peak response). On average, brighter stimuli had shorter latencies. (**D**) Average threshold-crossing latencies of the responses of functional patches (all of the patches and 75% of patches with shorter latencies are shown separately. The latencies are similar to those recorded from the LGN multi-units). (**E**) Average latency to peak response of functional patches. Brighter flashes had longer response latencies, in contrast to LGN multiunit responses. All values are given as mean and standard error.

First, responses with short latencies between 60 ms and 100 ms most likely reflect electrophysiologically recorded firing responses of RGCs; these were phasic responses with either ON or OFF preferences (N = 18). A second set of responses were observed within 100 ms to 1200 ms, consistent with calcium influx and/or cell microscopic swelling (N = 17). The slowest response component at 500–1200 ms latency showed the best correlation with the simultaneously recorded heart beat (N = 7). We therefore attributed those signals to retinal blood vessels. The magnitude of the maximum evoked response within each patch increased with flash brightness (Fig. 4B). Bleach onset and offset responses (2 s of 5.1 log cd/m^2^) were of similar magnitude but ON responses were more frequent and, on average, more prominent than OFF responses.

Multiunit recording data from the LGN are shown in Fig. 4C. The latency of threshold crossing in functional patches (response onset, 30 to 180 ms) was very sensitive to noise, but showed a similar trend to LGN units in that it reduced as the stimulus intensity increased. By contract, the latency to the peak optical response (Fig. 4E), increased with stimulus intensity. The ERG signal latency for these stimuli^25^ remained relatively constant at 20 ms for a-wave peak and 42 ms for x-wave peak (the slow b-wave, characteristic to rods-dominated rodent ERG is completely absent in tree shrew). The peak amplitude of both the a- and the x- waves increased linearly in the tested stimuli range (from 50 μV to 100 μV for the a-wave and from 200–300 μV to 500 μV for the x-wave). A similar tendency was seen with the simultaneously recorded multiunit LGN (Fig. 4A). The peak firing rate increased almost 4-fold from dim stimulus to the bleach; the sustained response during stimulus presentation was much smaller, but showed a similar trend.

**Figure 5:**
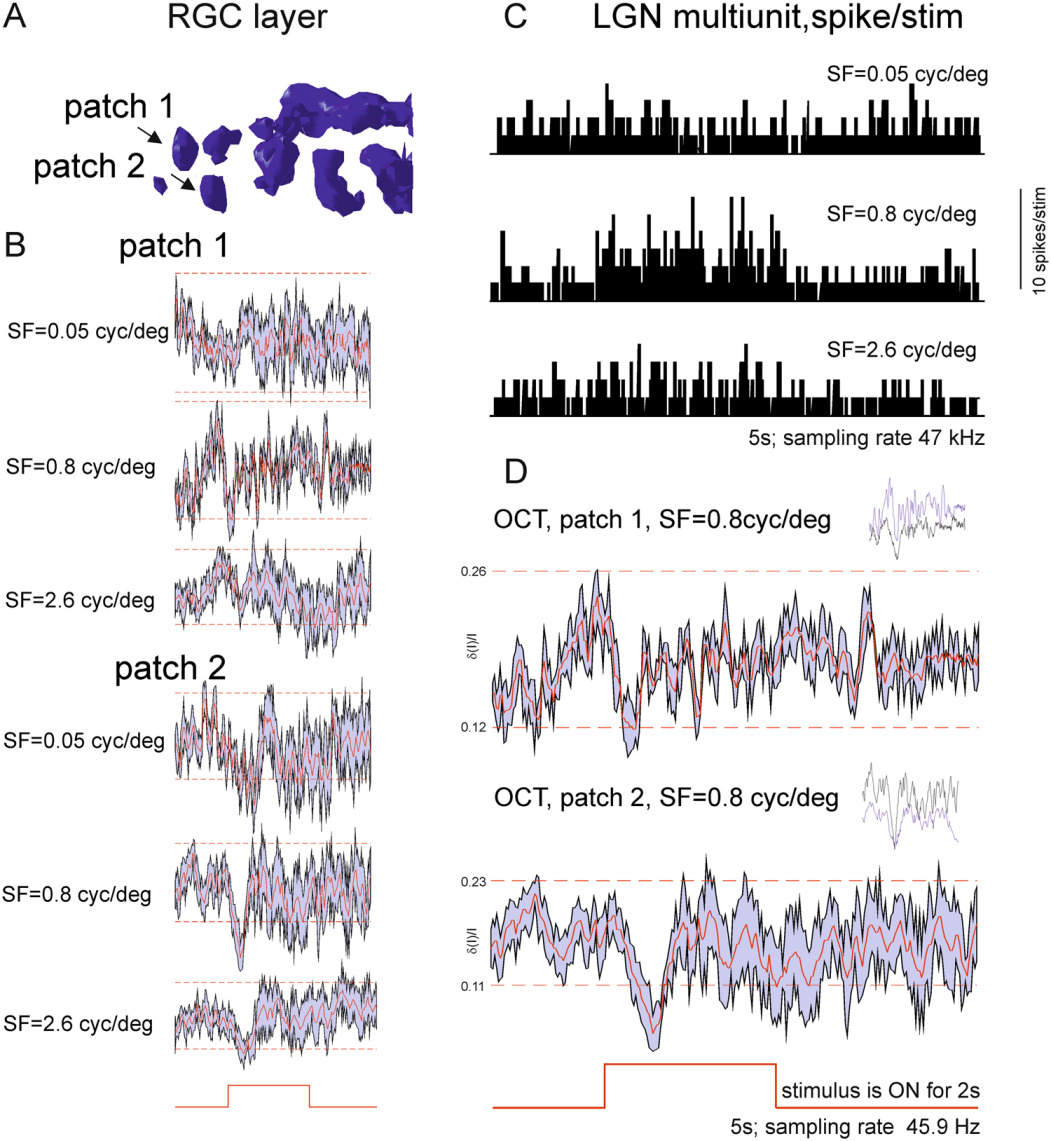
Patch responses to the moving gratings and correlation with LGN MUA. (**A**) An equal surface image of the RGC layer showing locations of two typical patches (**B**) Examples of responses of patches 1 and 2 to 100% contrast drifting (2 Hz) gratings of SF 0.05, 0.8 and 2.6 cycles/deg. The computer monitor stimulus was presented for 2 s, similar to flash stimuli. A grey screen was presented pre- and post- stimulus. The traces represent averages over several trials and over all pixels in the patch. Running error is given as maximum observed deviation of signal from the mean for a given time point and red dashed line represents significance threshold of 3 standard deviations from the trials with no stimulus presentation. (**C**) LGN MUA responses to drifting gratings of the same three SFs as in **B**, showing the strongest response at 0.8 cyc/deg (PSTHs with 20 ms bins). Two simultaneously recorded RGC patches responded to the same grating SF of 0.8 cyc/deg (the same traces as in B, magnified). Inset on the right shows trial-to-trial variability of average patch response.

To determine the relationship between patch responses and LGN multi-unit activity, we next presented drifting grating stimuli (in two tree shrews). Examples of responses from two RGC layer patches are shown in Fig. 5. We presented drifting gratings at 2 Hz and observed strong responses from two RGC patches and LGN MUA at a spatial frequency of 0.8 cycles/deg (responses were determined for frequencies in the range 0.01 to 6.4 cycles/deg). The responses were attenuated for lower spatial frequencies (0.05 cyc/deg) and higher special frequency (2.6 cycles/degree) that lie well within tree shrew’s spatial frequency range^26^. Given the duration of the experiments, we were only able to record responses for a limited number of spatial and temporal frequencies and were not therefore, able to derive a full spatial frequency-tuning curve.

Since the algebraic sum of the optical responses within a given region of interest will reduce the overall response, high-resolution images and the elimination of eye movements were essential for the differentiation of positive and negative changes in adjacent locations within the inner retina.

## Discussion

We report non-invasive assessment of inner retinal responses, with near cellular resolution, *in vivo* using OCT. To the best of our knowledge this is the first attempt to quantify inner retina optical response and separate different sources of the OCT signal at this resolution. Our work indicates that the quantitative optophysiological characterisation of stimulus-evoked responses in inner retina is technically feasible. The statistics of the observed response types matches closely to observed distribution of electrophysiological responses in tree shrew optic tract originally reported by van Dongen *et al*.^27^. However, the combination of electric and metabolic optical responses and the sensitivity of the optical signal to physiological fluctuation within the retina *in vivo* present major technical challenges and a significant barrier to the clinical application of this technology.

Several aspects of the custom OCT device are noteworthy. The use of a light source with long wavelength prevented accidental stimulation of retina with visible light, minimised loss of signal due to light scattering in cornea, and allowed optical penetration that would span the thickness of the human/primate retina. In contrast to confocal microscopy, the axial and transverse OCT resolution are decoupled. The axial resolution (4.87 μm, in this study) was determined by the optical bandwidth of the light source and the transverse (lateral) resolution (3.65 μm) by the optical aberrations of the imaging setup. The transverse resolution could further be improved with use of adaptive optics^28^.

The evoked signals seen in the outer retina in response to a brief flash were consistent with recordings from the photoreceptor layer as reported by Bizheva^12^. The slow negative response observed in the photoreceptor layer was similar to intrinsic signals obtained by fundus camera by Suzuki^22^.

The inner retinal responses increased in line with the intensity of the light flashes. We observed fast evoked responses in inner retina in relation to individual “patches” but, similar to Moayed *et al*.^23^, did not detect reliable responses in relation to the group average. However, the average increase in variability of the signal following the flash stimulus was consistent, suggesting that multiple patterns and response polarities in the inner retina may cancel each other out when averaged. To overcome this limitation we focused our analyses on inner retinal “patches” identified using a cluster segmentation algorithm. Simultaneous electrophysiological recordings from local retinal ganglion cells afferents allowed the objective evaluation of stimulus efficacy and a direct comparison of optical and electrical components of RGC responses. The short latency optical responses were comparable with “average firing rate” responses of RGCs. The long-latency responses matched slower intrinsic signals obtained by a fundus camera^22^ attributed to vascular changes.

The major movement artefact in OCT imaging comes from *micro saccades*. Even when gaze is fixed these small eye movements randomly shift the area of retina imaged by OCT. These movements are problematic for functional imaging of small signals where a small retinal area has to be imaged repeatedly. In addition, the small size of the region of interest precluded the use of re-alignment algorithms. For our study, the complete elimination of eye movement by systematic paralysis was essential.

The second source of movement artefacts came from *respiration*. This is more significant for animal studies compared to human studies, since humans can breath hold for the duration of the scan (ca. 5 seconds). In our experiment respiration was artificially controlled so that breath holding could be mimicked by “gating” acquisition - pausing artificial respiration during the OCT scan. In principle, respiration, when recorded simultaneously, has a distinct slow waveform and could be removed using signal processing.

Elimination of these two major sources of retinal movements simplified the task of movement compensation. Retinal pulsation due to the inner retinal circulation generated most of the retinal movement. We used subpixel registration of the images to compensate for physical oscillations and digital signal filtering for functional oscillations in pixel intensity. These are simple procedures that significantly reduced (but did not completely eliminate) “motion artefacts”. We did not attempt to account for the possible effects of arteriolar dilation/constriction due to stimulating light or glia signals^29,30^. Indeed, some of those movements, such as blood vessel shift or microstructural changes in vessel walls could have generated the slow signals observed in the inner retina in response to light stimulation. In addition, neurons (and cells in general) tend to change their size and position as a function of their electrical and metabolic activity. This could be reflected in their OCT-imaged profile and account for part of OCT-measured functional signal^9^.

Our results show that optical “functional” patches within the inner retina can either increase or decrease their optical intensity in response to the visual stimuli. The algebraic summation of these responses would reduce the apparent magnitude of the response when data are pooled over a wider area. Similar “punctate” regions with slow signals of either positive or negative polarity were reported (but not quantified) in inner retina^22^. In the present study, the optophysiological response “patches” correlated with the mean firing rate of local RGCs afferents and their scale approximated cellular components such as RGCs.

Low coherence imaging is sensitive to backscattered light (exceeding 100 dB). This can be an advantage, but it also makes it difficult to separate neuronal activation from light scatter, polarisation and reduced light transmission that have been observed following neuronal activation. The optical detection of neuronal activity has been reported by others but usually in atypically large neurons (e.g. giant squid axons^9^ or in isolated neuronal groups, e.g. in Aplysia^31^ where the optical changes are thought to reflect change in the alignment of membrane dipoles. The magnitude of the light response is proportional to the change in the membrane potential^32^. We have addressed the problem of discriminating multiple cell responses *in vivo* within a large neuronal population where the returned signal will be affected by the angle of the laser light on neurite or blood vessels^10,33,34^. Furthermore, *in vivo*, cell membrane depolarisation arises not just from action potentials but also from glia activation, metabolic changes in the mitochondria, and light-induced isomerization of molecules^11^. By comparison, the increase in the optophysiological signal in the outer retinal layers is striking. The limited microvasculature, packing density, alignment and uniform response polarity would all contribute to this effect.

The challenges in discriminating RGC-related responses in the inner retina *in vivo* currently limit usage of OCT in assessing RGC function in humans. Not surprisingly, attempts to record functional changes by OCT in humans have met with limited success^18,35–37^. With greater spatial and time resolution OCT (swept-source OCT and femtosecond laser OCT) improved characterisation of RGC responses should be possible without muscle immobilisation, possibly by using 3-D eye movement compensation algorithms^23,38^ and adaptive optics^39–41^. Our data provide the critical “proof of principle” regarding optophysiological functional characterisation of RGC using a non-invasive imaging modality with potential value for development of early retinal diagnostics.

## Methods

We constructed a high-resolution custom optical coherence tomography (OCT) with long wavelength light source (Fig. S1). The OCT comprised a built-in optical stimulator synchronised to record, simultaneously, OCT, stimuli, and physiological parameters, such as neuronal spiking activity, heart beat (ECG), cortex activity - electrocorticogram (ECoG), and respiration rate, all with the same sampling rate. The optical portion of the OCT device consisted of a turnkey amplified spontaneous emission light source (ASE,1-M-ASE-HPE-S, NP Photonics, Tucson AZ, USA) with a centre wavelength of 1040 nm and bandwidth (FWHM) of 70 nm connected to custom built objective optics and spectrometer via an 80:20 fiber-beam-splitter (FOSC-64-100-20-L-S-H-2, AFW Technologies, Hallam, Australia). The custom built objective optics moved a 0.5 mm radius OCT beam angled through the dilated pupil (~4 mm^2^). The imaging was raster-scanned using coupled galvanometer mirrors (6210HB, Cambridge Technology, Lexington MA, USA) to image 32 degrees of retina. The spectrometer had a cooled gallium arsenide linear array CCD (charge-coupled device) camera operating at 47 kHz (SUI1024LDH-1.7RT-0500/LC, Goodrich Sensors Unlimited, Princeton NJ, USA) connected via communication protocol Camera Link to a PC mounted frame grabber card (PCIe-1427, National Instruments). For maximum speed, the camera was allowed to operate in a free running mode. The speed of camera was the major limiting factor for the temporal resolution of our study. Though much faster cameras are now used in experimental OCT systems, the speed of our camera was much higher than in commercially available OCT systems and comparable if not superior to other OCT systems actively used for pre-clinical research and method development. A field programmable gate array (FPGA), (PCI-7833R 3 M, National Instruments, Newbury, UK) triggered by the camera frame pulses, was used to calculate the scan pattern in real time. The scan angle was calculated and provided as a row voltage output by the FPGA then passed to the digital galvanometer controller (Cambridge Technology, UK). The axial resolution of the system was estimated as 4.87 μm and lateral resolution as 3.65 μm (see the Appendix for the details) allowing the acquisition of retinal images at cellular resolution.

### Animal preparation

All experiments were undertaken in accordance with the UK Animals (Scientific Procedures) Act and the Association for Research in Vision and Ophthalmology (ARVO) guidelines for the use of animals in research. The experimental protocols were approved by Cardiff University Biological Standards Committee; Project Licence 30/2264. We followed the guidelines of the Animal and Plant Health Agency for compliance with Regulation (EC) 1069/2009 and its implementing Regulation (EC) 142/2011 for the transport, storage, use and disposal of animal by-products.

Adult tree shrews (Tupaia belangeri, n = 8, both sexes, 120–300 g, the youngest 175 days old), were anaesthetised for surgery with an intramuscular injection of ketamine (Vetalar, 50 mg/ml, 0.1 ml/100 g body weight) and xylazine (10 mg/ml, 0.1 ml/100 g body weight), followed immediately by the intramuscular injection of atropine (0.3 mg/kg) to minimize oropharyngeal secretions. Animals were fitted with a tracheotomy tube and femoral vein cannula, catheterized, and transferred to a stereotaxic frame. The anaesthesia was subsequently maintained by inhalation of 1.2–2.0% isoflurane in a mixture of 40% oxygen and 60% N_2_O for the duration of the experiment. The body temperature was maintained at 37 C. The heart rate was continuously monitored by electrocardiogram (ECG) and maintained in the range 200–270 beats/min. The depth of anaesthesia was monitored by electrocorticogram (ECoG) whereby isoflurane dosage was adjusted to maintain slow waves EEG punctuated with brief periods of cortical silence indicative of relatively deep sleep.

We employed electrophysiology as a “gold standard” measure of retinal ganglion cell activation in response to visual stimuli. To this end, a circular craniotomy (2 mm diameter) was performed above the left temporal cortex and the opening filled with warm saline. A single extracellular recording electrode (Melanie Ainsworth, Welford, UK) was inserted into the left lateral geniculate nucleus (LGN) to monitor the activity of axonal projections of RGCs from the contralateral (right) eye. After obtaining a stable neurophysiological response from the axonal afferents corresponding to RGCs in the area of retina accessible to OCT imaging, the animal’s muscles were immobilised by bolus i.v. injection of the gallamine triethiodide (initially 40 mg/kg IM, followed by 10 mg/kg/hour) to stop eye movements and allow rapid repetitive imaging of the same retinal segment. Respiration was maintained by a mechanical pump (RUS 1301, Föhr Medical Instruments, Germany) and was monitored with a clinical gas monitor (Datex-Ohmega RGM 5250 CO_2_ SpO_2_ Monitor, GE Healthcare, UK) the end tidal CO_2_ maintained in the range 3.2–3.6%. The pupil of the eye was dilated with atropine (1% eye drops) and the cornea was protected from drying with eye gel (Visgel) and a custom-made zero power gas-permeable contact lens (Cantor and Nissel, Brackley, UK). The optical quality of the eye was periodically monitored using a hand held ophthalmoscope. The position of the electrode in the brain was verified by histology. We also performed selective morphological assessment of retinal ganglion cells using electron microscopy to rule out any possible damage caused by the intensity of “bleach” flash stimuli.

### Imaging procedure

Several large 32 degree retina morphological B-scans (see Fig. S2A) were taken by OCT to confirm optical clarity, retinal health, and image stability (no saccades and drifting eye movements). Then a reliable multiunit electrophysiological signal was obtained from LGN afferents corresponding to RGC(s) in the area of retina accessible to OCT imaging (due to set-up limitations OCT had a range of mobility ~50 degree that covered central and peripheral retina). The receptive fields of the cells and their exact position on the retina were determined using an audio monitor, movable flat (LCD) computer monitor, and OCT attached stimulating LED light, focused at the same point on the retina as the measurement beam of OCT to allow the selective stimulation of the imaged retinal area. These arrangements ensured that some of the cells imaged by OCT were also captured electro-physiologically. A small area in central retina was then selected for optophysiological imaging with care taken to avoid major blood vessels (Fig. S2B). An additional large anatomical scan centred on the chosen area was then taken to establish exact anatomical coordinates of the OCT retinal recording (Fig. S2B). The selected area (32 by 32 pixels, 25 μm × 25 μm) was then imaged repeatedly for ~5 s (20 ms for a B scan). We used sub-resolution imaging with the size of pixels (0.77 × 0.77 × 1.84 μm) being smaller than the actual spatial resolution of the optical device (3.65 μm × 3.65 μm × 4.87 μm). The inclusion of more pixels minimised image distortion due to pixelation. The time resolution was limited by the camera speed (47 kHz), at approximately 20 ms/vol. Altogether, 256 volume scans were acquired, with 2 s stimulus presentation starting at volume 50. We used two types of visual stimuli – square 100% contrast moving gratings presented for 2 s on the computer screen (temporal frequency 2–3 Hz, spatial frequency 0.01–6.4 cycles/degree, generated using ViSaGe stimulus generator, Cambridge Research Systems, UK) and long-lasting (2 s) flashes of variable intensity (4 levels from dim (on the threshold of eliciting RGC electrophysiological response) to 98% bleach, at levels of corneal luminous flux 0.002, 0.014, 0044, 0.1 lm) generated by LED light (PW14 K2 Luxeon Star, Philips LUMILEDS, San Jose CA) driven by a high power LED driver (LEDD1B, Thorlabs, Newton NJ) attached to the OCT. Photo bleach values for the flashes were estimated from the values that would be achieved for a human eye (cone opsin only, 95% of tree shrew photoreceptors are cones), when exposed to similar luminance and flash duration.

The equation for the fraction *B* of pigment bleached at the end of the exposure of duration *t* at a retinal luminance *I*:

1 $$B=\frac{I}{I+{I}_{0}}(1-{e}^{-t(1+\frac{I}{{I}_{0}})/\tau }$$

where corneal luminance ca 1.6 × 105 cd/m^2^ and effective photopic retinal illuminance ca 3 × 106 Td.

Corneal luminous flux ignores the solid angle and does not take into account the size of the image on the retina. We thus calculated the retinal illuminance in cd/m^2^: 3.38 log cd/m^2^, 4.21 log cd/m^2^, 4.73 log cd/m^2^, and 5.12 log cd/m^2^. The Log (base 10) scale brings the values close to the perceptual scale of brightness.

Recovery of visual sensitivity in the dark after bleaching is limited by the regenerating rate of photo pigment (opsin in our case). The following equation describes the relation between pigment regeneration in the dark for a large variety of flash intensities (from 10 to 100% bleach of photo-pigment):

2 $$\mathrm{log}(\frac{Et}{Ea})=\alpha (1-\rho )$$

where *E*_*t*_ is an intensity of bleaching flash, *E*_*a*_ is a value of *E*_*t*_ found after dark adaptation, *p* is a fraction of pigment present and *α* is a constant associated with a particular pigment.

All experiments were conducted in a dark room to accelerate the recovery of bleached pigment. We presented a series of intermediate LED flashes (2 s each at various luminance) with the brightest flash bleaching ~98% of opsin. We allowed up to 50 minutes recovery time in the dark between the bleach trials. The time constant for cone dark adaptation is about 105 ms. Based on human data α is ~3.5. There is a small additional “neural” component to the retina recovery that is relevant for the first few minutes if weak flashes are presented in rapid succession. To eliminate this confounding factor we had sufficient time (at least 5 min) between the subsequent presentations of weak flashes to allow pigment recovery. Animals were dark adapted for at least 30 minutes prior grating stimuli presentation, and for at least 30 minutes between stimuli repetitions, if presentations were repeated in the same animal.

### Data analyses

All data were pre-processed using custom Matlab (Mathworks) software. First, we compensated for chromatic dispersion produced by the vitreous body of the eye as a by-product of the broadband laser that was used to increase axial resolution. All individual B-scans were isolated from the 5 s long acquisition file and stored separately. Within each volume scan, OCT data were separated into forward scanning and backward scanning halves to eliminate potential distortions arising from scanning pattern. Small displacements due to circulatory and breathing artefacts were compensated by subpixel image registration. All images were flattened at the location of the inner limiting membrane (ILM, the boundary between the retina and the vitreous body, formed by astrocytes) for inner retina analyses, and of the bright OS/RPE boundary, the landmark that was used to combine data from different experiments and analyse outer retina. The position of this latter boundary was calculated for the mean B-scan in the set and applied uniformly to all B-scans. The first five volumes without stimulus were averaged and used as a baseline background to subtract from subsequent data sets. These background-subtracted sets were run as movies to observe the qualitative character of the data.

After image registration of the B-scans, no discernible axial motion could be detected, but some artefacts of cardiovascular rhythm remained as speckle movement internal to the B-scan. A cardiac rhythm filter was applied; then a first order Savitsky-Golay filter (length 5 frames) applied in the time dimension to smooth rapid temporal fluctuations. Then, the differential OCT signal and its variance were calculated across time dimension of the scan.

The differential OCT signals, Δ(*x*, *z*, *t*) and its *variance* were obtained by:

3 $${\rm{\Delta }}(x,\,z,\,t)=\frac{R(x,z,t)-\langle {R}_{o}(x,z)\rangle }{\langle {R}_{o}(x,z)\rangle }$$

4 $$var(x,\,z,\,t)=\frac{{(R(x,z,t)-\langle {R}_{o}(x,z,t)\rangle )}^{2}}{\langle {R}_{o}(x,z,t)\rangle }$$

where, *R(x*, *z*, *t)* is the reflectivity at the position *x* and *z* at time *t*; and 〈*R*_0_*(x*, *z)*〉 is the average reflectivity *before* the stimulus (first 5 volumes) at the position *x* and *z*.

The changes in the reflectivity in each layer and its variance were evaluated by averaging the pixel values of several A-lines based on the layer segmentation of the retina (between the surface and the landmark OS/RPE boundary – the local maximum in the reflectiveness). The surface of the retina was defined at the level of two standard deviations from the background signal in the eye vitreous when moving in axial direction. To obtain the time course of the OCT signals, the results of 5 to 8 experiments were averaged.

We segmented the volume of inner retina further into “patches” based on the intrinsic similarity of pixel differential responses (measured by cross-correlation)^42^ (Fig. 3). The pixels in a “patch” were similar with respect to polarity and time course of stimulus evoked changes, while adjacent “patches” were significantly different with respect to the same characteristics. Due to sub-resolution imaging the image components had “shady” boundaries, for this reason only pixels with clear “patch” preferences (value of cross-correlation with neighbouring pixel above the automatically chosen threshold) were considered for the further analyses. We applied several iterations of Otsu’s segmentation^43^ method to automatically perform clustering-based image thresholding. This iterative method finds the threshold that minimises the variance within the “patch”, and maximises the variance between neighbouring “patches”. The resulting “patches” were consistent between stimuli for a given area (see Supplementary video data) of the retina and could be traced consistently throughout experiment. The “patches” were visualised in 3D and analysed separately. The polarity of the responses, and their time courses varied from “patch” to patch”. The “patches” were usually of oval shape and averaged to 5–8 pixels in diameter (5–20 μm). Based on the size, they may correspond to individual cells or segments of the blood vessels within the inner retina.

## Electronic supplementary material

Supplementary Appendix and Figures
