# Supplementary material for: Optophysiological Characterisation of Inner Retina Responses with High-Resolution Optical Coherence Tomography
Source: Sci Rep. 2018 Jan 29;8:1813. doi: 10.1038/s41598-018-19975-x (PMC5788978; doi:10.1038/s41598-018-19975-x)
Supplement: Supplementary file 1 — Supplementary Appendix and Figures [file 41598_2018_19975_MOESM1_ESM.pdf]

Supplementary materials

Optophysiological Characterisation of Inner Retina Responses with High-Resolution Optical Coherence Tomography

Irina Erchova[1], Alexandre R. Tumlinson[1,5], James Fergusson[1], Nick White[1], Wolfgang Drexler[4], Frank Sengpiel[2], and James E Morgan[1,3*].

[1]School of Optometry and Visual Sciences, Cardiff University, Cardiff, United Kingdom;

[2]School of Biosciences, Cardiff University, Cardiff, United Kingdom;

[3]Ophthalmology, University Hospital of Wales, Cardiff, United Kingdom;

[4]Center for Medical Physics & Biom Eng, Medical University Vienna, Vienna, Austria

[5]Zeiss Meditec, Dublin, California, USA;

*Address for correspondence: School of Optometry and Visual Sciences, Cardiff University, Maindy Rd, Cardiff, CF24 4HQ

Correspondence to MorganJE3@cardiff.ac.uk

Supplementary figures legends

Figure S1.

OCT design. The light from the laser source (NP photonics < 17mW @ 1040± 35nm, energy stability 1%, beam divergence ~15deg from source, collimated after fibre to 2mm) is transmitted via fibre-optics (input fibre coupler (fc)) and split into measuring and reference beams via a 2 x 2 80:20 fibre splitter (fs). Three outputs from the fibre-splitter deliver this light to imaging device (20%, fibre coupler (fc), scanning mirrors (sm), telecentric lens pairs (tl), short pass dichroic mirror (dm)), reference arm (80%, input fibre coupler (fc), adjustable retro reflector (rr)) with variable path retro-reflecting back to the spectrometer, and to spectrometer (input fibre coupler (fc), gold spherical mirrors (gm), diffraction grating (dg), line scan digital camera (c)), measuring

interference between direct and reflected light. The imaging device projects light into the eye and collects reflected light signal that is first compared to the reference signal, and recorded by digital camera.

Figure S2.

Recorded data. A. Large anatomical OCT scan of 36 degree of tree shrew retina centred on small region of interest (ROI) in the receptive field of LGN MUA. B. Reconstruction of retinal blood vessels from the scan (A) with optic nerve head (ONH) located just outside the image on the right. The small ROI of 32x32 pixels is in the central retina away from major blood vessels. C. Summary of all recorded locations in relation to ONH.

Figure S3.

Heartbeat artefact. A. The heartbeat represented a major challenge in respect to both retinal micro movements (as explained in Figure 1) and variation in the strength of the OCT signal. The OCT (corrected for all type of movements) signal intensity of four example traces (right) from four different retinal strata (regions marked on OCT intensity profile (left) in relation to simultaneously recorded heartbeat. B. An additional caveat was that our volume sampling frequency was similar to tree shrew heart rate which created a low “beat” frequency, potentially masking stimulus responses. Black line represents a down-sampled heartbeat trace. We were able to effectively reduce heart beat contamination of the response signal by applying digital filters in the frequency domain (blue line). Inevitably, this procedure led to distortion of the signal in time domain, as shown by red line.

Figure S4.

Size comparison between functional “patches” and image texture “clusters”. The image texture was dominated by short noise (mostly 1-4 pixels size). The functional “patches” were on average larger and were of the same size as RGC cells of inner retina blood vessels.

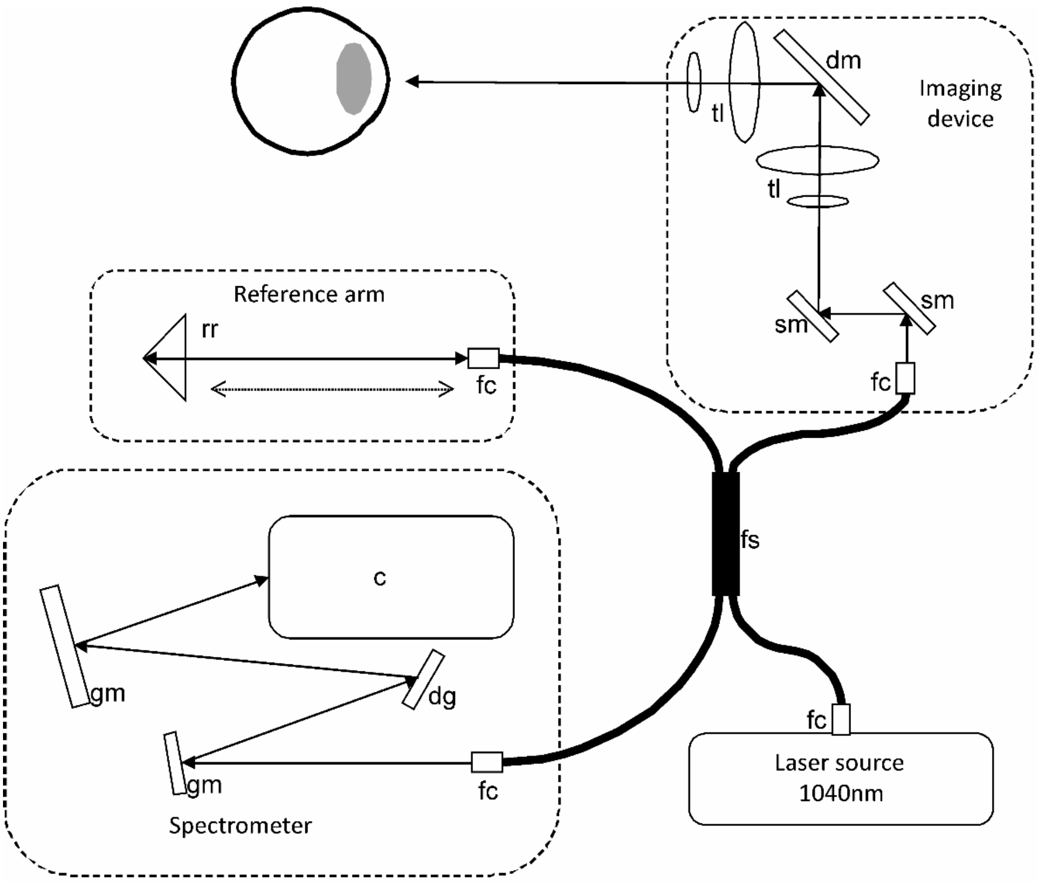
dm
Imaging
device
tl
tl
Reference arm
rr
fc
sm
sm
fc
fs
c
gm
dg
gm
fc
fc
Laser source
1040nm
Spectrometer

Figure S1

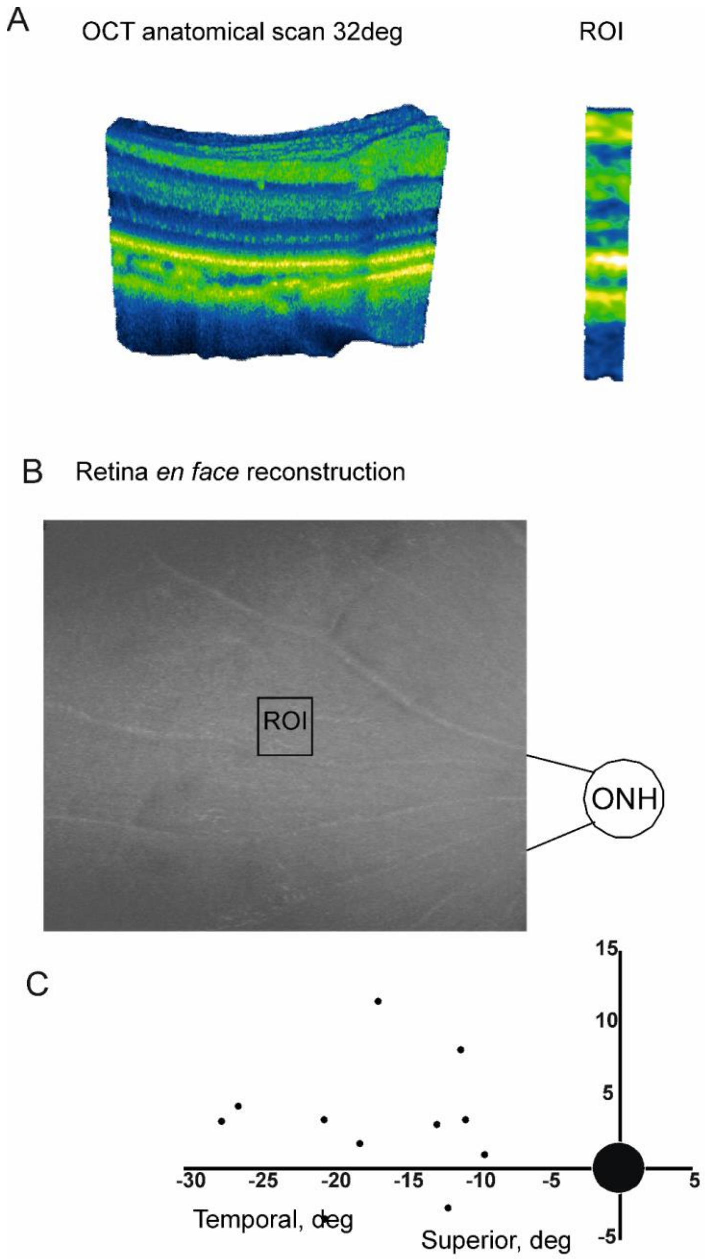
A
OCT anatomical scan 32deg
ROI
B
Retina en face reconstruction
ROI
ONH
C
15
10
5
-5
-30
-25
-20
-15
-10
-5
5
Temporal, deg
Superior, deg

Figure S2

A Heartbeat artefact across retinal layers

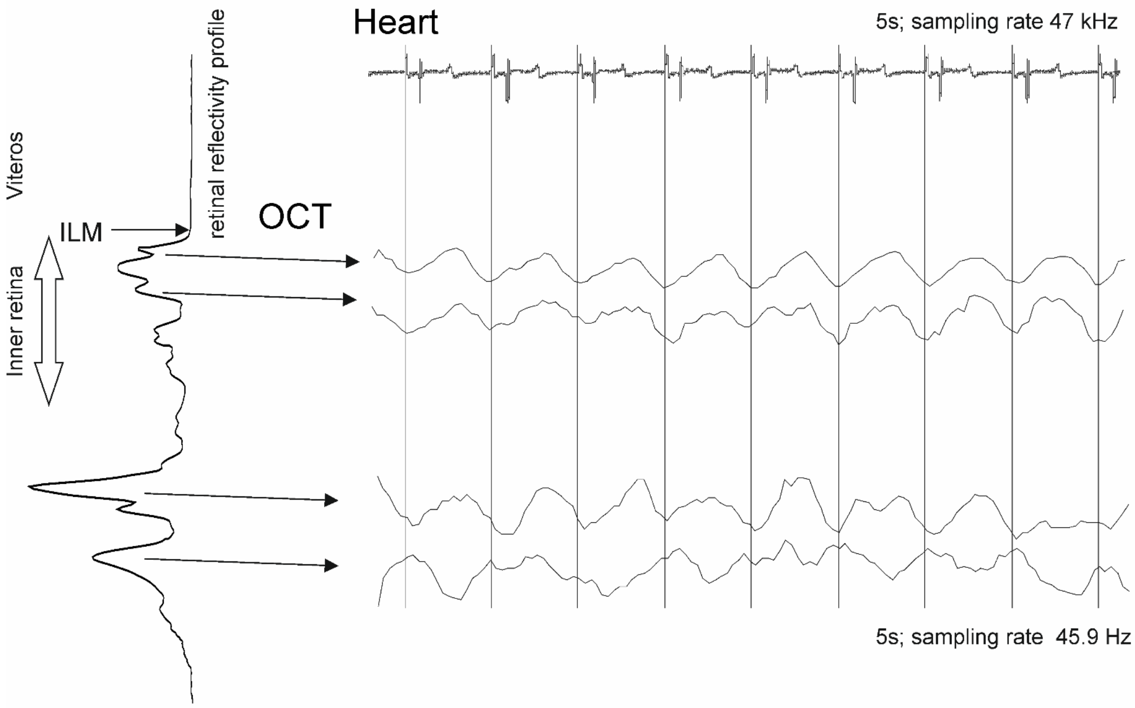

B Filtering out heartbeat artefact distorts time of stimulus response

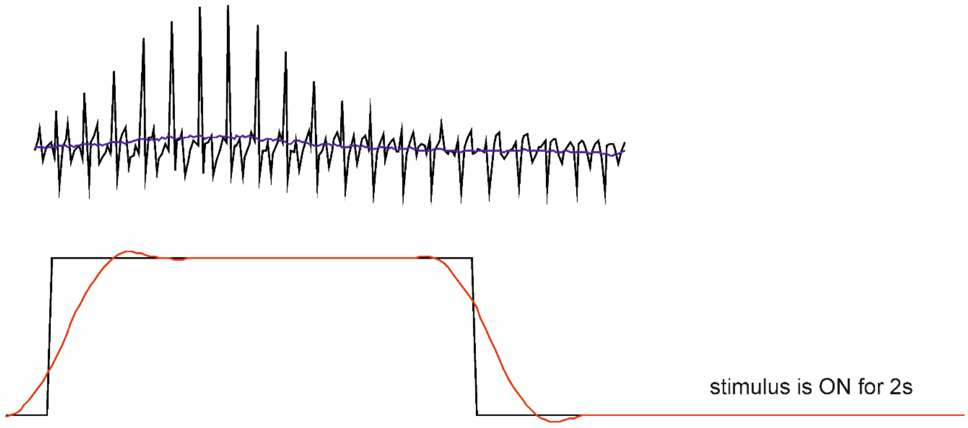

Figure S3

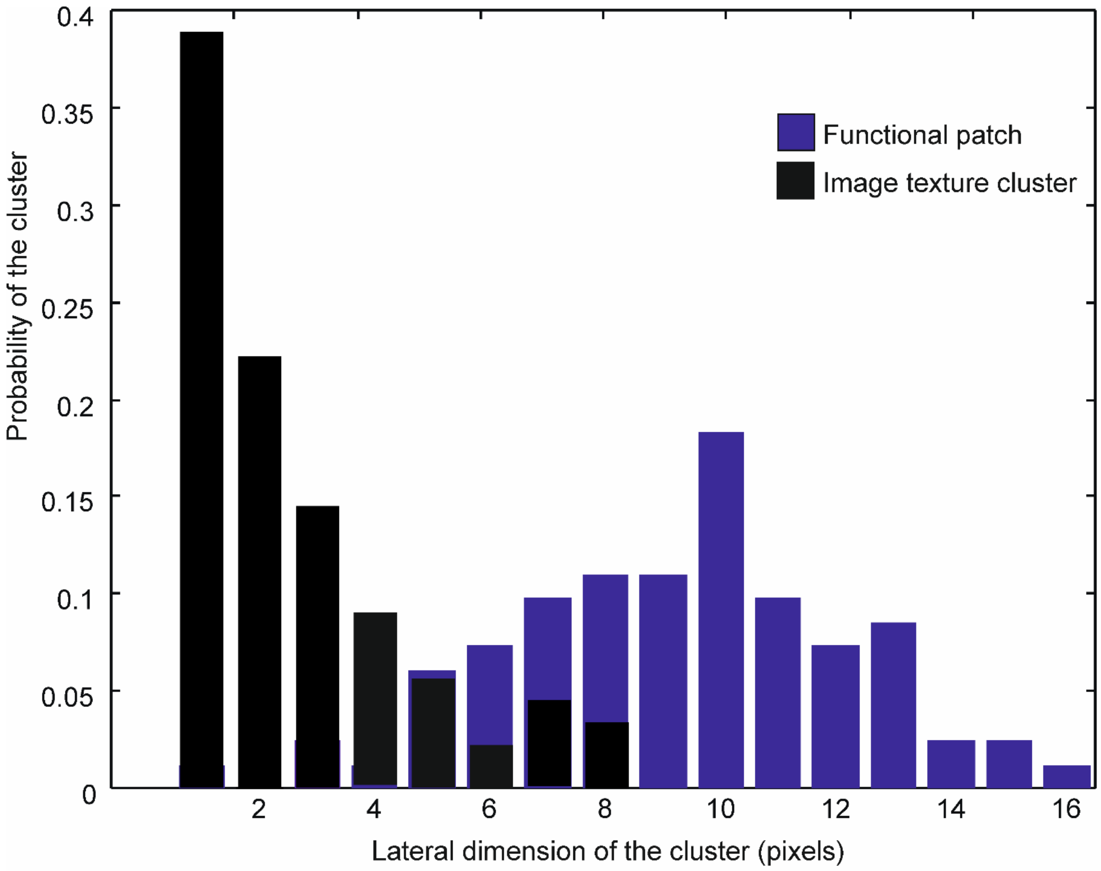

0.4
0.35
0.3
0.25
0.2
0.15
0.1
0.05
0
Probability of the cluster
Functional patch
Image texture cluster
2
4
6
8
10
12
14
16
Lateral dimension of the cluster (pixels)

Figure S4

Appendix: OCT resolution and scaling calculations

The interferometric properties of optical coherence tomography result in a decoupling of the transverse and axial resolutions (at a given wavelength), depending on the optics of the instrument and the design of the spectrometer respectively. The transverse and axial scaling are also different, the former being a property of the lateral sampling rate and the latter being a property of the instrument. However, both are also dependant on the sample that is being investigated. Our OCT system has been designed exclusively to look into the eye; it produces a collimated beam that must be focussed in order for a sharp image to be created. Therefore both the lateral scaling and lateral resolution depend on the optics of the eye being imaged. The axial scaling and resolution are only affected by the refractive index of the eye. The parameters of tree shrew eye where either measured experimentally or taken from published report by Norton and McBrien (1992).

Lateral Resolution

Lateral resolution was estimated using theoretical formula for Confocal Scanning Laser Ophtalmoscope (CSLO).

$\delta_L = \frac{0.4\lambda}{NA}$ (*Eq. A1*),

Where λ (λ=1040□m) is the central wavelength of the light source, and NA is the numerical aperture of the lens in tree shrew eye.

$NA = n * sin(\Theta)$ *(Eq.A2)*,

Where *n* (*n* =1.336) is the refractive index of tree shrew's eye. For the posterior focal length of the tree shrew's eye L_{pos} (L_{pos}=5.81mm) and an OCT beam radius of 0.5mm, Θ was calculated to be approximately 4.92^o, and thus NA=0.115.

The resulting theoretical *Lateral Resolution* is estimated as 3.65 μm. The actual *Lateral Resolution* for a particular image may deviate due to individual differences in tree shrew's eye size.

Lateral Scaling

The lateral scaling for the functional OCT images (0.3 degree scans) was calculated as following:

$L_S = \frac{2\pi R(\frac{A}{360})}{N_p}$ *(Eq. A3),*

Where *R* (*R*= 4.61 mm for 75 days old tree shrew) is the retina to nodal point distance. *A* is the size of the image in degrees; and N_p is the number of pixels in the scan (N_p=32). The lateral scaling was estimated at 0.75 μm. To take in account natural variability of the tree shrew axial eye length, we model tree shrew's eye in WinLens 3D software (Qioptic).

For the *Lateral Scaling* we used most conservative estimate of 0.77 μm.

Axial Resolution

Axial resolution, being a property of the instrument can be directly calculated.

$$\delta_A = \frac{2ln2}{\pi n}\frac{\lambda}{\Delta\lambda} \text{(Eq. A4)}$$

Where λ (λ=1040nm) is the central wavelength of the light source, and $\Delta\lambda$ ($\Delta\lambda = 70\ nm$) bandwidth (FWHM) of OCT (as before).

The *Axial Resolution* (in air) was 6.8 μm, and *in retinal tissue 4.87 μm* (taking in account retinal tissue refractive index *n* (*n*= 1.4).

Axial Scaling

Axial scaling was estimated empirically. We placed an object of known size in the sample arm and then measured its size in pixels from the resulting image. The target object was a glass clover slide suspended 1mm above a standard glass slide. Using a confocal microscope the air gap between the two slides was measured to an accuracy of 1μm. The same target was imaged with OCT and the scaling calculated as the air gap size/number of pixels on the image.

The axial scaling for our system in air was ~2.578 (±0.005) μm/pixel. For any other medium the axial scaling is

$$L_A = \frac{2.578}{n} \text{(Eq. A5)}$$

Where *n* is the refractive index of the sample.

The *Axial Scaling in retinal tissue was 1.84μm* (taking in account retinal tissue refractive index *n* (*n*= 1.4).
